# Non-local real-space analysis of chiral optical signals

**DOI:** 10.1039/c6sc01743f

**Published:** 2016-07-11

**Authors:** Jérémy R. Rouxel, Vladimir Y. Chernyak, Shaul Mukamel

**Affiliations:** a Department of Chemistry and Department of Physics and Astronomy , University of California , Irvine , California 92697-2025 , USA . Email: jrouxel@uci.edu ; Email: smukamel@uci.edu; b Department of Chemistry , Wayne State University , 5101 Cass Ave , Detroit , Michigan 48202 , USA . Email: chernyak@chem.wayne.edu

## Abstract

A spatially non-local response tensor description of linear chiral signals such as circular dichroism is developed. By working directly with the vector potential rather that the electric and magnetic fields, we recast the signals in terms of correlation functions of charge and current densities and avoid the tedious expansion in multipoles.

## Abstract 2

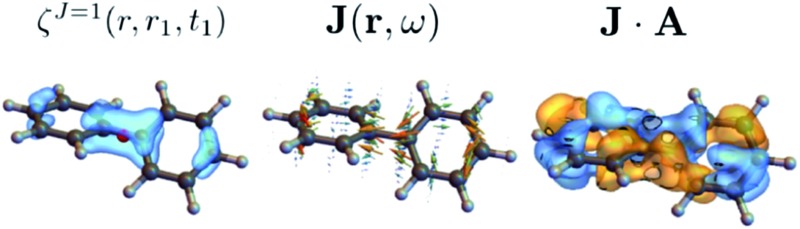
A spatially non-local response tensor description of linear chiral signals such as circular dichroism is developed.

Circular dichroism (CD), the difference in absorption of right and left circularly polarized light, has long been used to distinguish chiral objects (enantiomers) which lack a mirror symmetry in their point group.^1^ Recently, a proposal by Tang and Cohen^2^ has triggered an intense activity in understanding and enhancing such enantioselective signals for plane waves which are intrinsically weaker than non chiral signals^3–5^ by a factor *a*/*λ* where *a* is a molecule size and *λ* the optical wavelength, this factor is typically 10^–2^ to 10^–3^. The circular dichroism signal is defined as the difference of the signals from two circularly polarized plane waves, a LCPL (left circularly polarized light) and a RCPL (right CPL):1
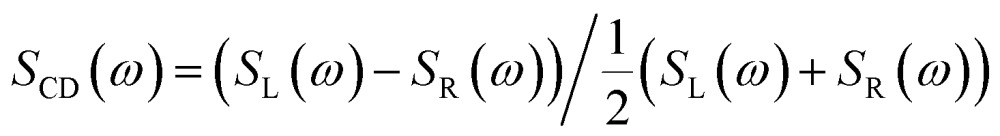
with *S*_L_(*ω*) and *S*_R_(*ω*) being the absorption of left and right CPL at frequency *ω* respectively.

Under some assumptions, Tang and Cohen have shown that *S*_CD_ can be factorized into a product of the chirality of the matter and the local chirality of the field: *S*_CD_ = (*R*/|**p**|^2^)(*C*/*ωU*_e_). The material quantity that creates the CD signal depends on the rotatory strength *R* = Im(**p**·**m**) of the molecule, where **p** and **m** are the electric and magnetic dipoles respectively. *U*_e_ is the time average electric energy density and *C* is the optical field chirality 
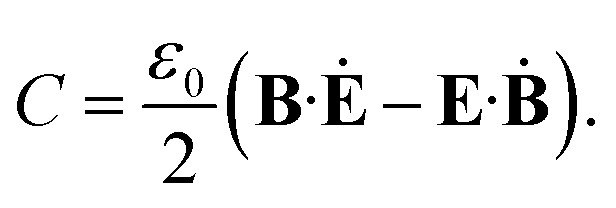


This relationship has permitted the design of hyperchiral fields that amplify chiral signals beyond the *a*/*λ* factor that applies to plane waves. However, this simple picture is limited to the electric and magnetic dipole approximation of matter which may be insufficient when working with fields that strongly vary across a molecule or a nanostructure.^6,7^ Moreover, optimizing the local field chirality *C* creates an enhanced chirality only at one point, where the electric field amplitude is small.

Standard CD experiments that use plane wave fields only exploit the handedness of the field through its spin degree of freedom, *i.e.* its polarization. Coles and Andrews^4^ have shown that the optical chirality density *C* is only a measure of the spin angular momentum of the field. We have recently developed a more general non-local approach that includes all higher multipoles and accounts for the full spatial variation of the exciting fields over the space of the molecule. This is simpler than the multipole expansion which can become tedious when high multipoles are needed. Also, while CD experiments are usually defined for a rotationally averaged sample and only reflect the lack of inversion symmetry, oriented samples of molecules or nanoplasmonic nanostructures^6–8^ can be studied as well. In that case, a CD signal can be obtained in both chiral and achiral systems and depends on the relative orientation of the exciting beams and the molecule.^1^ The nonlocal chiral approach allows the visualization of the physical quantities relevant for these interactions.

In this paper, we demonstrate that the transition current densities that naturally originate in the non-local response, as briefly discussed above, provide useful microscopic insights into the nature of optical response. In particular, it helps to identify the regions of a molecule where the response is created. These can further assist in developing experimental settings to measure how strong is the chiral nature of an achiral molecule.

In Section 1, we derive expressions for the chiral response that take into account the complete spatially extended interaction of the molecule with the incoming field and include the entire (orbital and spin) angular momentum of light. All the relevant quantities, discussed in Section 2, are physically meaningful because care is taken to keep gauge invariance at each step.^9^ The nonlocal representation may further help in optimizing enantioselective signals using spatially varying light.

## 1 The chiral non-local response functions

### 1.1 Irreducible non-local response functions

The multipolar Hamiltonian may be used in order to account for nonlocal field–matter interaction. The driving electric field then interacts with the position-dependent polarization 
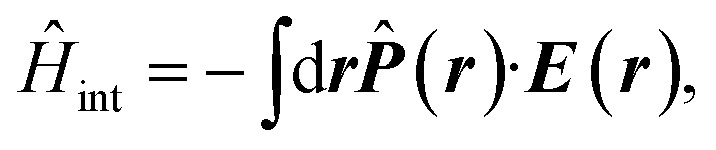
 ^16^ with the polarization operator being a non-local linear functional of the system charge density *ρ̂*(***r***), making the transition densities *ρ*_*αβ*_(***r***) = *α*|*ρ̂*(***r***)|*β* the key quantities that contain the complete information on the molecule nonlocal response. However, a simple picture of non-local response, described above is obtained only if the magnetic terms involving the current density operators ***ĵ***(***r***) are neglected. Otherwise, the multipolar formalism becomes cumbersome. This reflects the fact that, in quantum mechanics, the interaction between charges and electromagnetic fields can be written in terms of the scalar-vector potential (*A*_0_(***r***), ***A***(***r***)), which changes under gauge transformation, whereas all observables must be gauge invariant.^10^ This makes the problem of nonlocal optical response rather involved.

A gauge-invariant approach to the nonlinear responses has been developed^9^ recently that uses the quantum mechanical field–matter interaction, involving the scalar/vector potentials and charge/current densities, and treats the optical response functions as gauge-invariant functionals of the non-gauge-invariant scalar and vector potentials. Making use of gauge invariance of the response functions, one can fix a gauge, such that the induced matter polarization is expressed as an expansion in powers of the driving electric field, although the field matter interaction involves the vector potential, rather than the electric field. This combines the advantages of the traditional expansion in powers of driving electric field with the field–matter interaction being properly accounted for. This results in the explicit expression for the nonlocal response functions in terms of correlation functions of the charge and current density operators, which makes the transition charge and current densities the key ingredients of the response function that, together with the transition frequencies completely determine the non-local response. Since the continuity equation ∂*ρ̂* + **∇**·***ĵ*** = 0 is valid on the operator level, the transition charge densities can be explicitly expressed in terms of the current densities, making them the only necessary ingredients for the non-local responses.

We focus on optical signals given as the rate of change in the number of photons in the observed modes:2
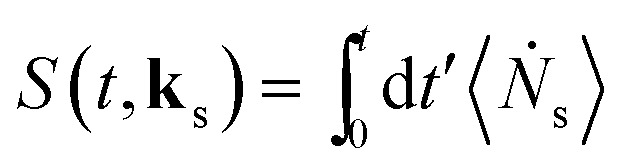
where the time dependence of *S*(*t*, **k**_s_) comes from the expectation value taken at time *t* and represented by …. The change in transmission of an incoming field, also known as heterodyne detected signal^11^ may be recast in the form:^9^
3
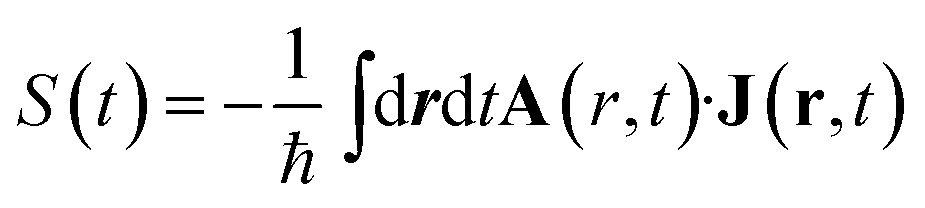
where **A** is the vector potential of the incoming field and **J** is the classical current density obtained as the expectation value of the gauge-invariant current density operator 
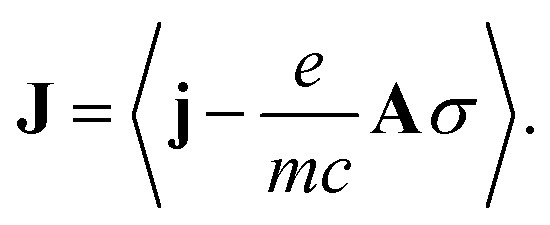
 **j** is the bare current density operator (in the absence of an external vector potential):4
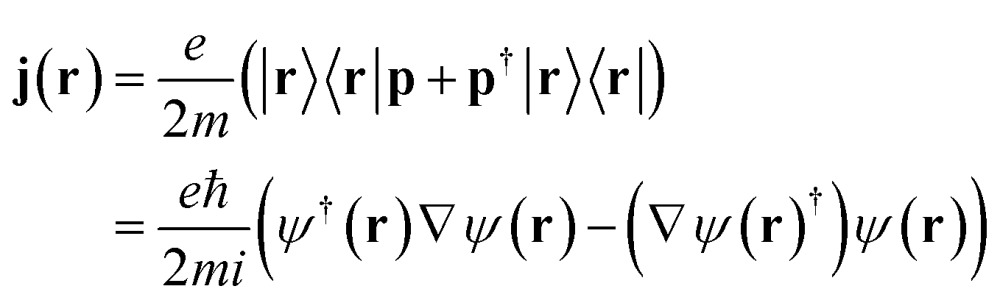

**p** is the momentum operator (**p** = **p** ^†^), *ψ* ^†^(**r**) and *ψ*(**r**) are the electron field creation and annihilation operators for an electron in position **r** and *σ*(**r**) = *eψ* ^†^(**r**)*ψ*(**r**) is the charge density operator.

Expressing **J**(**r**) in terms of the polarization **P**(**r**) and the magnetization **M**(**r**) densities^12^ (**J**(**r**) = ****(**r**) + ∇ × **M**(**r**)) recovers the standard local expression:5
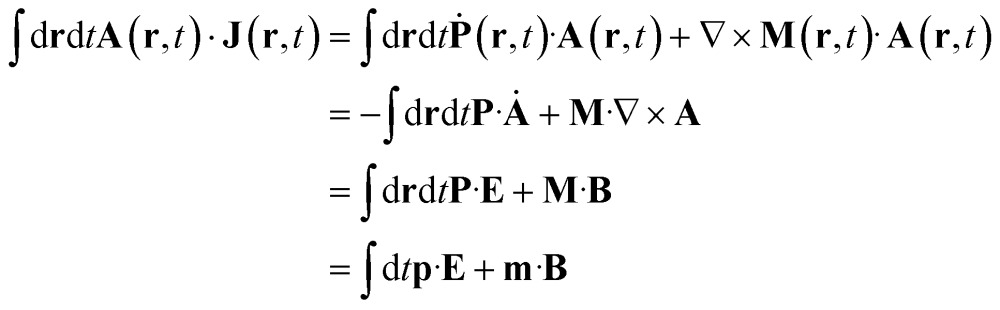
where **E** = –**** and **B** = ∇ × **A** are the electric and magnetic field respectively. Between the first two lines, we have used the relation **E**·∇ × **M** = (∇ × **E**)·**M** – ∇·(**E** × **M**); the second term vanishes as one can see using the Green–Ostrogradski theorem (volume integral of the divergence of a bounded distribution). To recover the dipolar approximation, one could consider fields that do not vary over the molecular space and define the electric and magnetic dipole moments expectation value 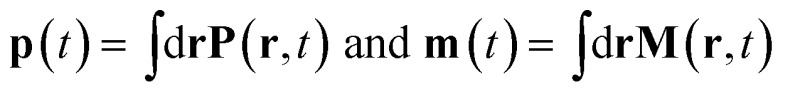 respectively.^9,13^Eqn (5) are only given here to connect with standard description of optical signals.^1^ We shall not use them hereafter. Our theory only uses the vector potential rather than the electric and magnetic fields, and the current and charge densities instead of the magnetization and polarization. The multipolar expansion, although useful when it can be truncated at low multipoles, becomes cumbersome when dealing with strongly spatially varying fields and does not provide the simple intuitive picture offered by the current density.

Specific linear and nonlinear signals can be obtained by expanding **J** perturbatively in the incoming vector potential **A**. We shall focus on the linear response6
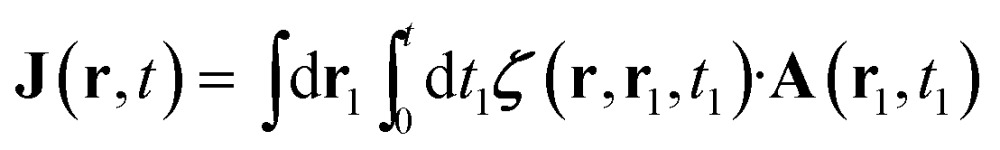
where ***ζ*** is the nonlocal linear response tensor whose Cartesian matrix elements are:7
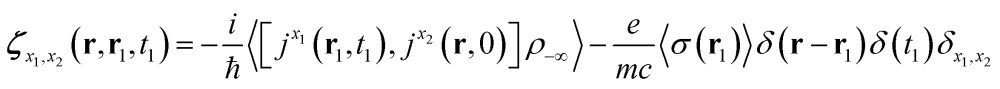
where *ρ*_–∞_ is the density matrix before interaction with the incoming beams. Inserting eqn (6) into (3) leads to:8
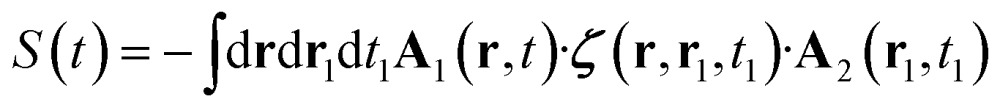



The subscripts 1 and 2 for the vector potential indicate that the heterodyne and the exciting fields can be different (for example, one is left and one is right handed circularly polarized). In standard CD experiment, **A**_1_ = **A**_2_ and the subscripts will be dropped henceforth.

Since the molecular chiral response is closely related to the angular momentum carried by the incoming light, an irreducible tensor formalism will prove convenient.^4,14,15^ At each point of its 6-dimensional (**r**, **r**_1_) space, the linear response tensor is expanded using an irreducible basis:

9 ***ζ*** = ***ζ***^*J*=0^ + ***ζ***^*J*=1^ + ***ζ***^*J*=2^

Each component transforms under rotation as an irreducible representation of the rotation group, care should be given to also rotate the variable as we are dealing with an irreducible tensor field. Eqn (8) can then be recast as:10
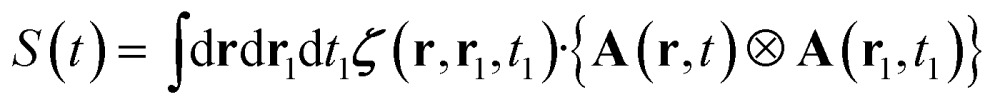



The dot products in eqn (8) have been replaced by a fully contracted product between the linear response tensor and the irreducible product of the vector potentials.^15^ Since irreducible tensors of different angular momentum are orthogonal, one gets the fully contracted product:11
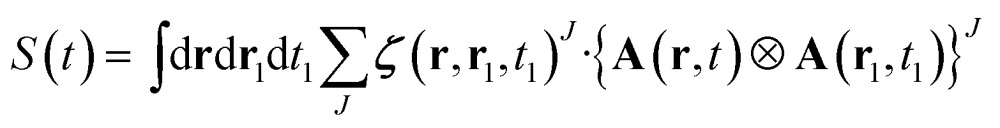



Chiral signals are associated with the *J* = 1 component of the irreducible response tensor because it is a pseudo-vector that lacks inversion symmetry. As will be shown in the next section, in a CD experiment, the incoming field interacts with the *J* = 2 components of the response tensor. Although this part is symmetric, it can still contribute to chiral signals. As stated by Varshalovich,^15^ an irreducible tensor of rank *n* can be split into an even and an odd tensor under inversion, corresponding to each connected component in O(3).

One can design experiments that solely probe the *J* = 1 component. This irreducible component can be recast as a cross product of the current densities. ***ζ*** ^*J*=1^ can be expressed as:12
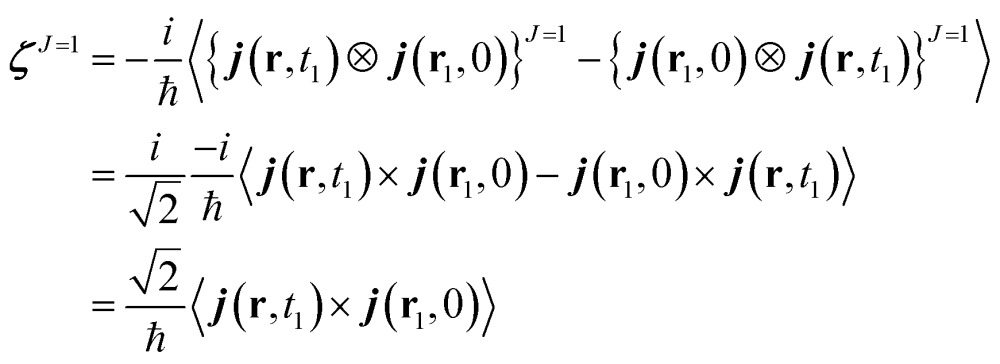
where {*a* ⊗ *b*}^*J*^ is the irreducible tensor product.^15^ In the last line, we have used the fact that *ε*_*lm*_^*k*^[*j* ^*l*^(**r**_1_, 0) ,  *j* ^*m*^(**r**, *t*_1_)] = 0 (summation implied).

The charge density does not appear here because it only contributes to the isotropic (*J* = 0) part of the response tensor. Being expressed as an antisymmetric product, ***ζ*** ^*J*=1^ is a pseudo vector which does not change sign under inversion of the coordinates system. Thus, all of its the components vanish if a molecule has a mirror symmetry (non chiral).

**Fig. 1:**
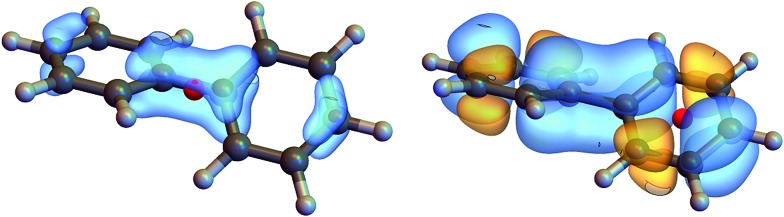
Contour plot of ***ζ*** ^*J*=1,*M*=0^(**r**, **r**_1_), eqn (12), for **r**_1_ at the chiral center (left column) and at the center of the benzene ring (right column). The position corresponding to the chosen value of **r**_1_ is depicted by a red dot. Other components *M* = 1 and *M* = –1 are not considered in this article since they interact with the longitudinal part of the incoming field.

We shall illustrate the nonlocal chiral response for biphenyl and hexahelicene (Fig. 1). Twisted biphenyl can be compared with its planar, non chiral, stereoisomer and thus serves as a convenient benchmark for chiral molecules. Excitation energies and transition current density matrix elements were calculated at the configuration interaction singles (CIS) level using the 6-31G* basis set. The *M* = 0 of the antisymmetric part of the response tensor for two different transitions in twisted biphenyl are displayed in Fig. 1.

The nonlocal formalism takes in account the entire spatial variation of the field over the molecule and the total (spin and orbital) momentum of light.^16^

### 1.2 Circular dichroism signals

The denominator of the CD signal, eqn (1), in the nonlocal formalism is:13
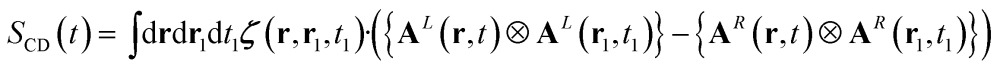



**Fig. 2:**
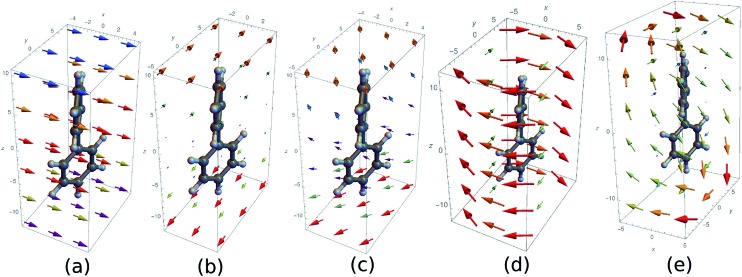
Several electric fields used to excite a biphenyl (the real part of the complex field is displayed). The vector colors indicates the norm of the vectors. (a) Plane wave polarized along *x* and wavelength 210 nm (b) superposition of a left and right polarized plane wave (c) superposition of LCPL and RCPL with a slightly different amplitude (corresponding to Cohen's proposal^5^) (d) azimuthally polarised beam (*λ* = 210 nm, *w*_0_ = 10*λ*) (e) superposition of two azimuthal beams propagating along *z* and *x*.

We shall discuss conventional CD experiments with incoming plane waves as well as with the hyperchiral field configuration proposed recently by Tang and Cohen^5^ to enhance the chiral response. Fig. 2 represents the electric vector field over a biphenyl molecule for various field configurations. At the excitation wavelength (210 nm), a plane wave field (a) is uniform. Fig. 2(c) represents the electric field corresponding to Cohen's proposal, a superposition of left and right circularly polarized light of slightly different amplitudes. This field varies appreciably across the molecule and a nonlocal approach is called for. Finally, we present the electric fields of an azimuthal beam (d) and a superposition of two azimuthal beams (e). These fields possess a total angular momentum originating from both their spin and orbital momenta. Their major drawback is that their amplitude is small around their focal point as in Cohen's proposal.

For conventional CD, we assume CPLs propagating in the *z* direction:14
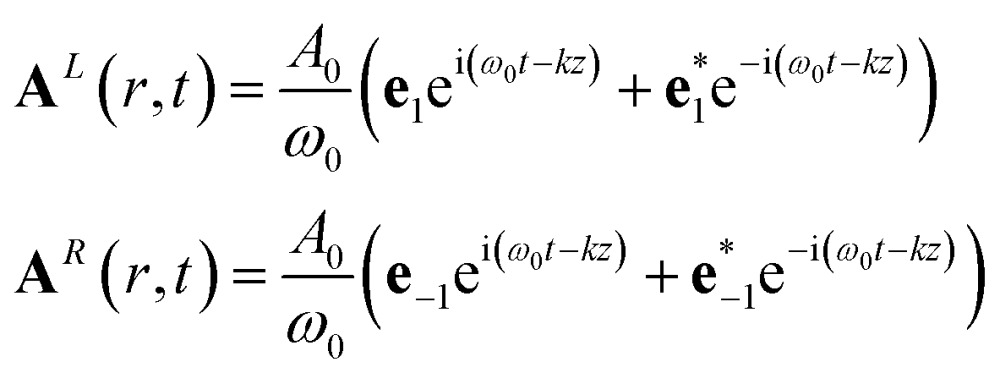



The division by *ω* gives the amplitude the units and value of the associated electric field.

Calculating the elements of the irreducible tensor products leads to several non-vanishing components: {**A** ^*L*^(**r**, *t*) ⊗ **A** ^*L*^(**r**_1_, *t*_1_)}^2,2^, {**A** ^*R*^(**r**, *t*) ⊗ **A** ^*R*^(**r**_1_, *t*_1_)}^2,–2^ and {**A** ^*R*^(**r**, *t*) ⊗ **A** ^*R*^(**r**_1_, *t*_1_)}^1,0^. The non vanishing components 2,0 and 0,0 cancel in the CD signal when taking the difference between left and right absorption. The denominator of the CD signal then becomes:15
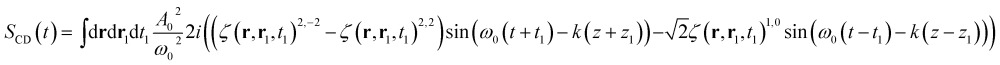



**Fig. 3:**
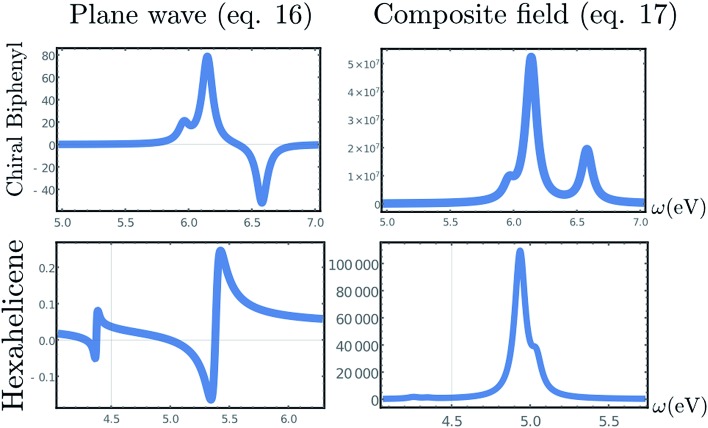
Top, Left: CD spectrum (eqn (1)) of a twisted biphenyl calculated using the non-local formalism, eqn (1)–(13). Rotational averaging has been carried out by rotating the incoming beam on a single molecule. Top, right: chiral signal obtained by the superposition of two CPL of slightly different amplitude, eqn (17). Bottom row: same as top but for hexahelicene.

The CD signals for the first four optical transitions of biphenyl and hexahelicene are given in Fig. 3. Since the spatial variation of the field across the molecule is weak, these results should coincide with the electric/magnetic dipole approximation.

Tang and Cohen^5^ have recently proposed to use a superposition of two counter propagating circular plane waves having a slightly different amplitude in order to maximize the chiral signal. The vector potential is then given by:16
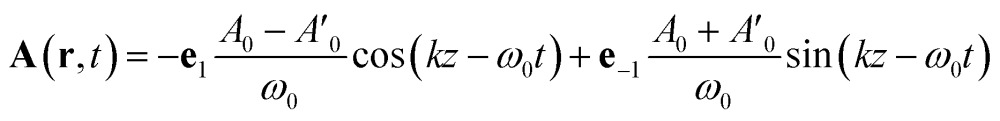



The non vanishing components of the reading field are:17
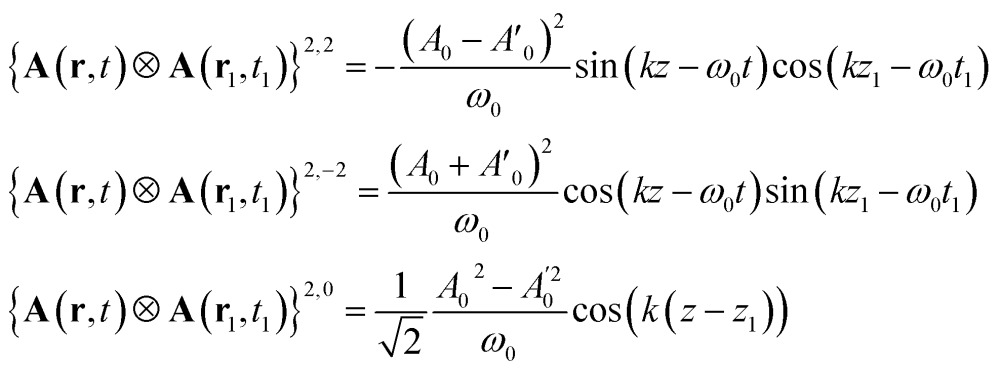



Contrary to the plane waves, the field contains a contribution *J* = 1, *M* = 0. However, this function is even in space and its contribution to the signal vanishes when integrated out with the antisymmetric part of the response function.

The computed signals (1) for biphenyl and hexahelicene are shown in Fig. 3. The molecule has been placed at the origin where the cosine function is maximal. The signal depends on the difference in amplitude *δA* between the two plane waves: as this difference becomes small, the signal is amplified as predicted by Cohen.^5^ However, as highlighted by Cohen^5^ and Cho,^3^ the signal enhancement is strong only when the molecule is positioned near the nodes of the exciting field.

## 2 Real-space visualization of the chiral response

The nonlocal formalism depends on the following physical quantities:

• The response tensor ***ζ***(**r**, **r**_1_, *t*_1_) (eqn (7)): this quantity contains all the relevant information about matter. An expansion over its irreducible components can highlight specifically the part of the response that is antisymmetric and leads to a chiral signal. The response tensor is mathematically a second rank tensor over a 6-dimensional space (**r**, **r**_1_) and one has to rely on slices of it to extract information as done in Fig. 1.

• The total current density **J**(**r**, *t*) (eqn (6)): the current density **J** is the sum of the currents generated by both the **j**·**A** and the *σ* **A** ^2^ couplings. This quantity can be directly interpreted as a classical motion of charge (expectation value of the gauge invariant current density operator).

• The **J**(**r**, *t*)·**A**(**r**, *t*) term is the integrand in eqn (8) which carries information regarding the combined system matter-field and describes the spatial origin of the measured signal.

**Fig. 4:**
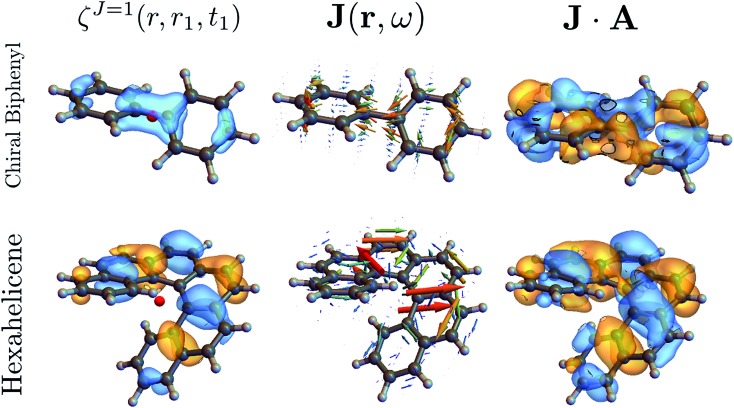
Component *M* = 0 of the antisymmetric part of the response tensor (left, eqn (7)), total current density (center, eqn (6)) and integrand **J**·**A** that generates the signal (right, eqn (8)) for twisted biphenyl (top) and hexahelicene (bottom). The antisymmetric part of the response tensor is calculated for the first transition (ground state to first electronic excited state). **J** and **J**·**A** are calculated from eqn (6) for an **A** field resonant with the first transition.

The above quantities are displayed for biphenyl and hexahelicene in Fig. 4. These molecules belong to the two typical classes of chiral molecules: one for which the mirror symmetry is broken at a specific spatial point and one where it is broken globally.

As can be seen in eqn (13), signals generated by currents at different parts of the molecule interfere to yield the overall signal. In addition, we can visualize which parts of the molecule contribute to a given signal. For example, chiral signals are generated in the vicinity of a chiral center as can be seen in Fig. 4 (biphenyl) while it is generated over the entire molecule in Fig. 4 (hexahelicene). Such valuable real space information is missed when using multipoles.

## 3 Discussion

In the electric and magnetic dipole approximation, the CD signal, eqn (1), is given by the rotatory strength.^17^ Being the product of a vector and a pseudo-vector, it must vanish under coordinate inversion for a non-chiral molecule that is invariant under coordinate inversion.

These considerations can be extended to the nonlocal representation but the parity of the fields that interact with the current densities must also be taken into account. In fact, although the irreducible tensor formalism permits to properly describe the spin part of the field angular momentum, which captures all the physics in the dipole approximation, it does not take into account its orbital angular momentum. Thus, a field that has, for example, a non-zero *J* = 1 component may still give a zero signal when integrated out with the *J* = 1 part of the response tensor if their spatial extension (*i.e.* their angular momenta in this case) are orthogonal. One could further expand the second rank tensor fields into bipolar vector spherical harmonics but this would involve the cumbersome multipolar algebra that was avoided so far. Moreover, such an expansion would be meaningful only if one were able to generate field that belongs to this basis, this is an interesting issue. Thus, the non-local formalism offers the proper level of irreducible expansion.

**Fig. 5:**
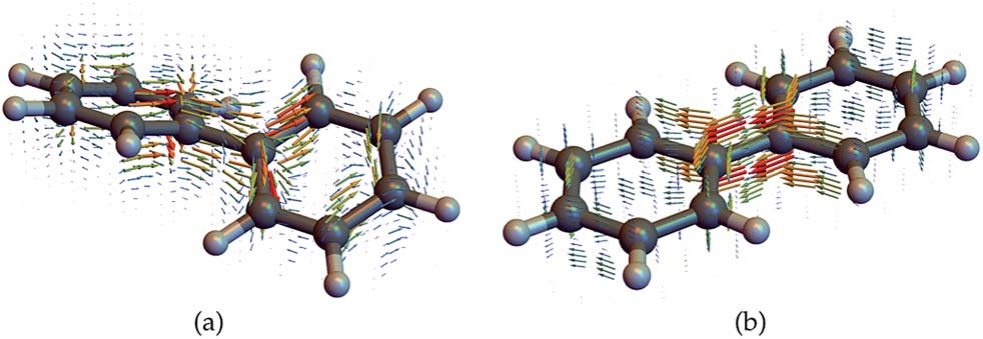
Transition current densities (eqn (4)) for the first excited state in (a) chiral twisted biphenyl and (b) non chiral planar biphenyl used to compute the response functions and CD signals.

We now display the current densities and demonstrate how they contribute to the nonlocal CD signals. To understand the interaction of a molecule with a chiral field, we need to consider how its transition current densities, as displayed in Fig. 5, project into the irreducible representations of the inversion group. Then, using a standard group theoretical picture, the vector potential field tensor and the response tensor must belong to the same (odd or even) representation in order to make a contribution to the signal that has been recast as an overlap integral. Additionally, many CD experiments involve the difference between various contributions, typically *ζ* ^2,2^ and *ζ* ^2,–2^, making the CD signal vanish for achiral system, even if each overlap integral may not vanish for given incoming fields. For example, the non-chiral (planar) biphenyl current density displayed in figure contributes equally to the *ζ* ^2,2^ and *ζ* ^2,–2^ components and will then cancel out in the signal (eqn (11)) when integrated out. In contrast, these components will add up for the chiral (twisted) biphenyl shown in Fig. 5a. Moreover, *ζ* ^1,0^ itself vanishes directly for the non-chiral case since the response tensor has no antisymmetric part if the system is invariant under parity. For the chiral case, it may contribute or not depending on field symmetry.

In summary, we have applied the non-local approach to study the chiral response of molecules. Our formalism takes into account the spatial variation of the exciting field across the molecule without relying on the multipolar expansion. While the latter has proven extremely useful for small systems in which only the lowest order terms are dominant, it becomes less tractable for highly focused nano-shaped pulse or near-field interactions. Also, working with multipolar matrix elements is less physically insightful than with the current densities. While the former correspond to an integrated components of a spherical expansion, the latter can be easily interpreted as a direction for the motion of charges.

The nonlocal formalism allows to treat traditional CD experiments as well as recently proposed hyperchiral light sources or other types of fields. This paper has focused on molecules. However, the approach can be readily applied to nanostructures for which nanoshaped fields are already of common use. In particular, one can readily think about experiments that use two different nanoshaped light sources **A**_1_(**r**, *t*) and **A**_2_(**r**, *t*) applied to single molecules or ensembles. The latter requires to deal with various averagings^18^ of the molecular tensor fields. Extensions to chiral nonlinear optics are possible as well.^9^

### 3.1 Electric and magnetic parts of the nonlocal response tensor

In order to connect with the standard treatment that uses the polarization **P** and the magnetization **M**, one can split the current density into its longitudinal and transverse parts using Helmholtz decomposition:

18 **J**(**r**, *t*) = **J**_*T*_(**r**, *t*) + **J**_*L*_(**r**, *t*)

19
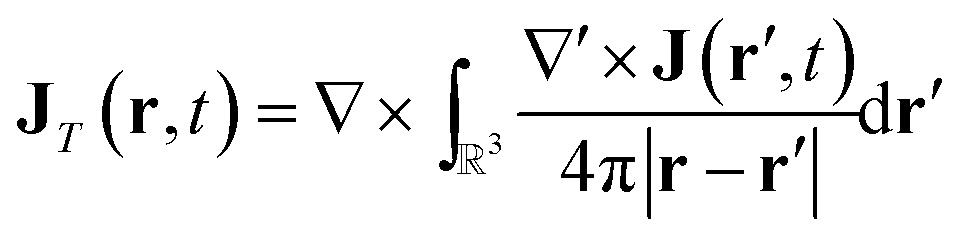

20
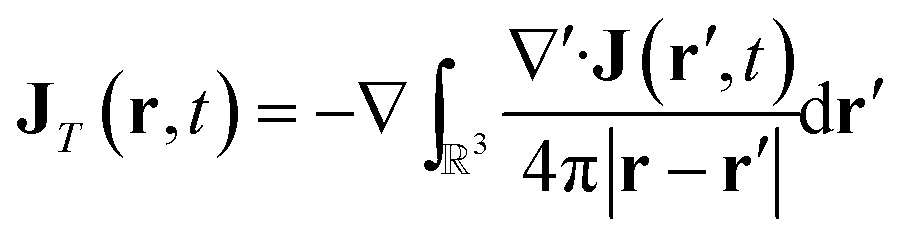



By comparison with the definition of **J** = **** + ∇ × **M**, we can express the polarization and the magnetization in terms of the linear nonlocal response function:21
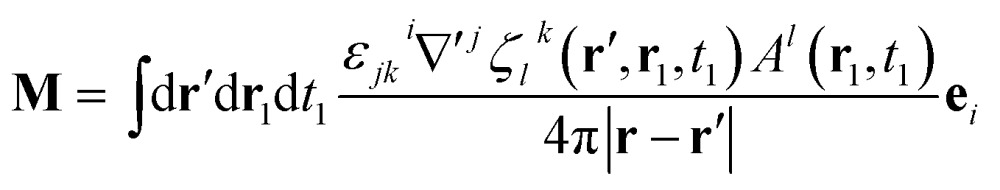

22
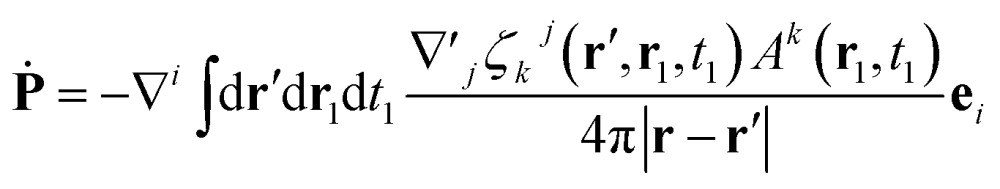



Magnetic and electric responses can be defined:23
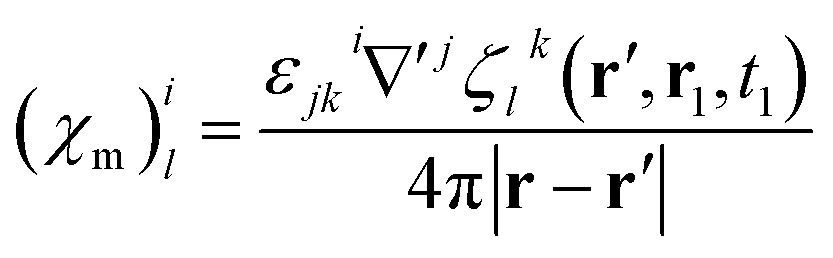

24
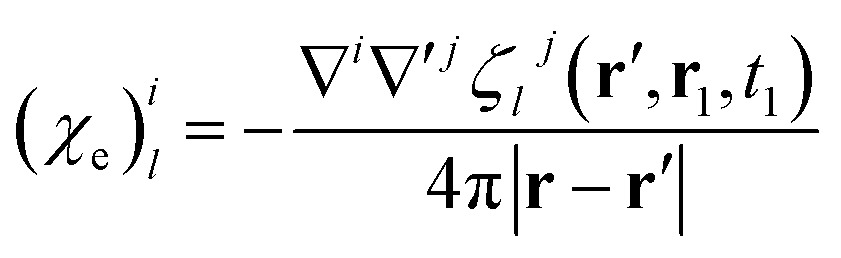



The magnetization and polarization are finally written in the following form:25
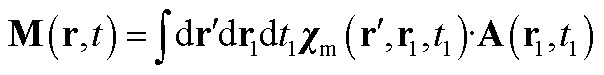

26
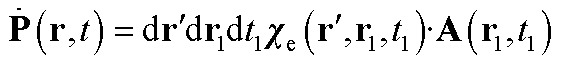



Note the extra integration over **r′** which is a consequence of expressing **M** and **** in term of the vector potential rather that **B** and **E**.

### 3.2 The irreducible tensor formalism

For completeness, we review briefly some properties irreducible tensors. A more detailed discussion about this formalism can be found in many references.^14,15,19^ Any Cartesian tensor can be expanded as a sum of irreducible tensors:27
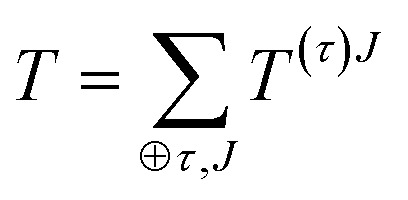
where *τ* is the seniority index^14^ used to distinguish different irreducible components of same *J*. In the irreducible expansion of a rank 2 Cartesian tensor, this index does not play a role.

Each irreducible component constitutes a vector space spawned by an irreducible representation of the rotation group SO(3), which mean by definition that a rotation is done through a Wigner *D* matrix:28
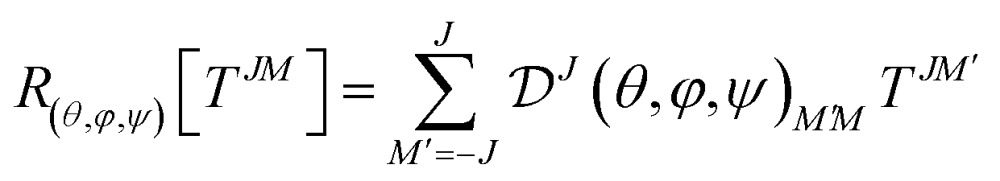



Since the angular momentum algebra is a special case of the irreducible tensor formalism, one recovers many similar expressions. In particular, the irreducible tensor product is defined as:

29 {*T*^*j*_1_^ ⊗ *U*^*j*_2_^}^*JM*^ = *C*_*j*_1_*m*_1_,*j*_2_*m*_2__^*JM*^*T*^*j*_1_*m*_1_^*U*^*j*_2_*m*_2_^

where *C*_*j*_1_*m*_1_,*j*_2_*m*_2__^*JM*^ is a Clebsch–Gordan coefficient. The produced tensor is an irreducible tensor of weigh *J*. The fully contracted product between tensor of same ranks is easy to define:30
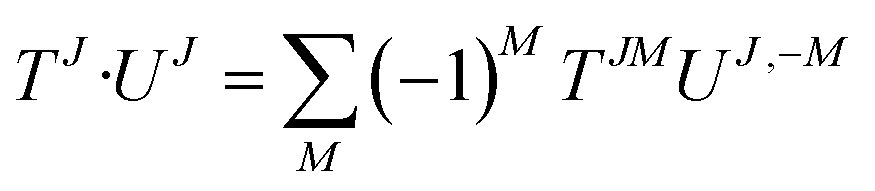



From this definition, one can see that the metric is antidiagonal of the form (–1, 1, –1). A vector of rank one has a straightforward expression as a *J* = 1 irreducible tensor. Its irreducible components are obtained through the following change of basis:31
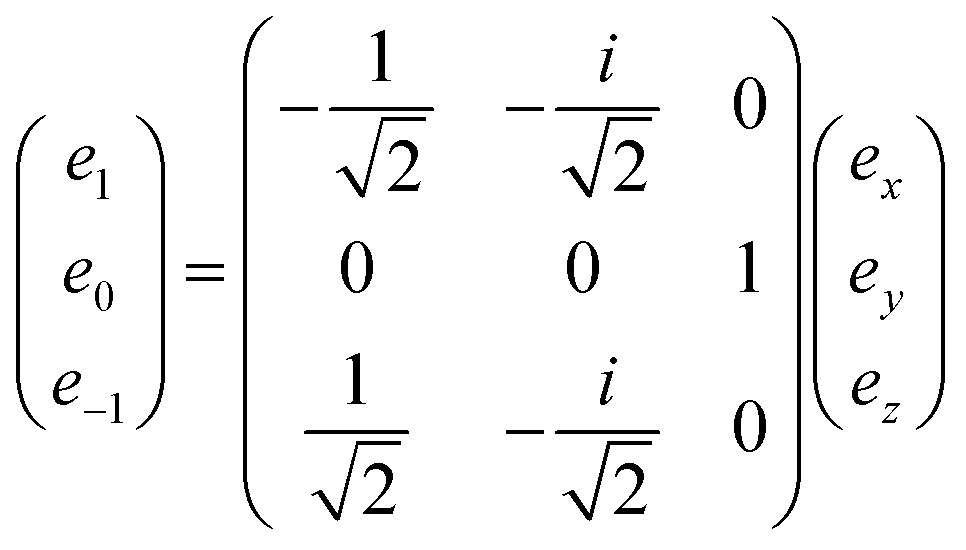



A Cartesian tensor of rank 2 is a direct sum of irreducible tensors of weight *J* = 0 1, 2. In the context of this article, it is useful to write the irreducible components of a rank 2 Cartesian tensor obtained from the direct product between two vectors in terms of standard vector algebra:32
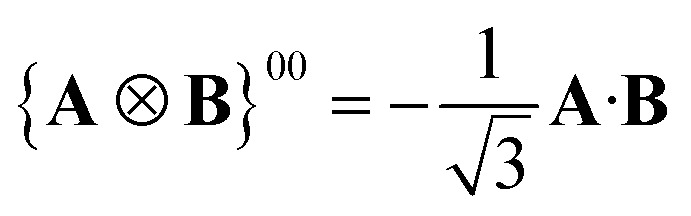

33
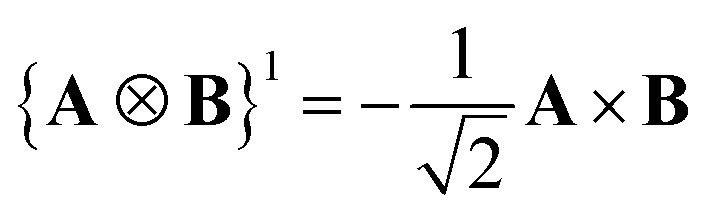

34
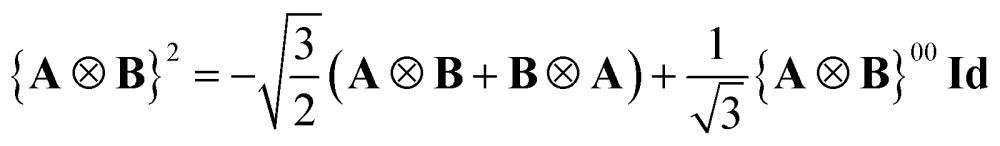
where **Id** is the identity matrix.
